# Extra-adrenal Pheochromocytoma of the Urinary Bladder in an Adolescent Female Patient

**DOI:** 10.7759/cureus.75785

**Published:** 2024-12-16

**Authors:** Kunal Bajaj, James Neibling, Pauline Do, Gili Edry, James W Davis

**Affiliations:** 1 Diagnostic Radiology, Detroit Medical Center, Wayne State University, Detroit, USA; 2 Pathology, Detroit Medical Center, Wayne State University, Detroit, USA; 3 Pediatric Radiology, Detroit Medical Center, Wayne State University, Detroit, USA

**Keywords:** bladder pheochromocytoma, extra-adrenal pheochromocytoma, paraganglioma, plasma metanephrine, urinary bladder pheochromocytoma

## Abstract

Pheochromocytoma is a catecholamine-secreting tumor that arises from the medullary chromaffin cells but can rarely be extra-adrenal in origin. We present a case of a 16-year-old female patient with uncontrolled hypertension, despite being on lisinopril and metoprolol, and associated left-sided chest pain, recurrent headaches, and an unintentional weight loss of 10 pounds in one month. Laboratory work-up showed a markedly elevated plasma metanephrine level of 4463.4 pmol/L (normal range <510 pmol/L). Since it is important to find the location, rule out malignant pheochromocytoma, and consider preoperative strategy, imaging studies were performed. Abdominal ultrasound revealed a hypervascular mass in the right adnexa, while an MRI of the abdomen and pelvis identified a lobulated soft tissue mass in the right hemipelvis with diffusion restriction that appeared to be arising from the urinary bladder. Given the clinical presentation, significant elevation in catecholamines, and MRI results, extra-adrenal pheochromocytoma (EAP) was the leading differential diagnosis. Phenoxybenzamine was initiated to control her blood pressure and a partial cystectomy was performed to excise the tumor involving the right posterolateral bladder wall. Histopathological report from urinary bladder tissue biopsy confirmed the diagnosis of EAP. This case highlights a rare and intriguing presentation of EAP arising from the urinary bladder, successfully managed with partial cystectomy.

## Introduction

Pheochromocytomas are neuroendocrine tumors with 80-85% of cases originating from adrenal medullary chromaffin cells and the rest being non-adrenal, with extra-adrenal pheochromocytoma (EAP) accounting for 10-15% in adults and 30-40% in children [1,2]. Urinary bladder paraganglioma is extremely rare, constituting less than 0.06% of all bladder tumors, with one-third of them not demonstrating any hormonal abnormality [3]. Initial workup for suspected EAP includes laboratory tests, including serum and urine catecholamines. Because bladder EAP is extremely rare, it is often overlooked. Performing surgery or cystoscopy without any prior imaging increases the risk of life-threatening hypertensive crises without anti-adrenergic medications. MRI is the optimal imaging technique for diagnosing EAP and establishing locoregional staging due to its superior soft tissue resolution and ability to distinguish between layers of the bladder wall and from other bladder masses [4].

In this report, we described a rare case of primary pheochromocytoma with classical symptomatology and an unusual location of origin from the urinary bladder. The expression of chromogranin A and synaptophysin were consistent with the diagnosis of pheochromocytoma.

## Case presentation

**Figure 1:**
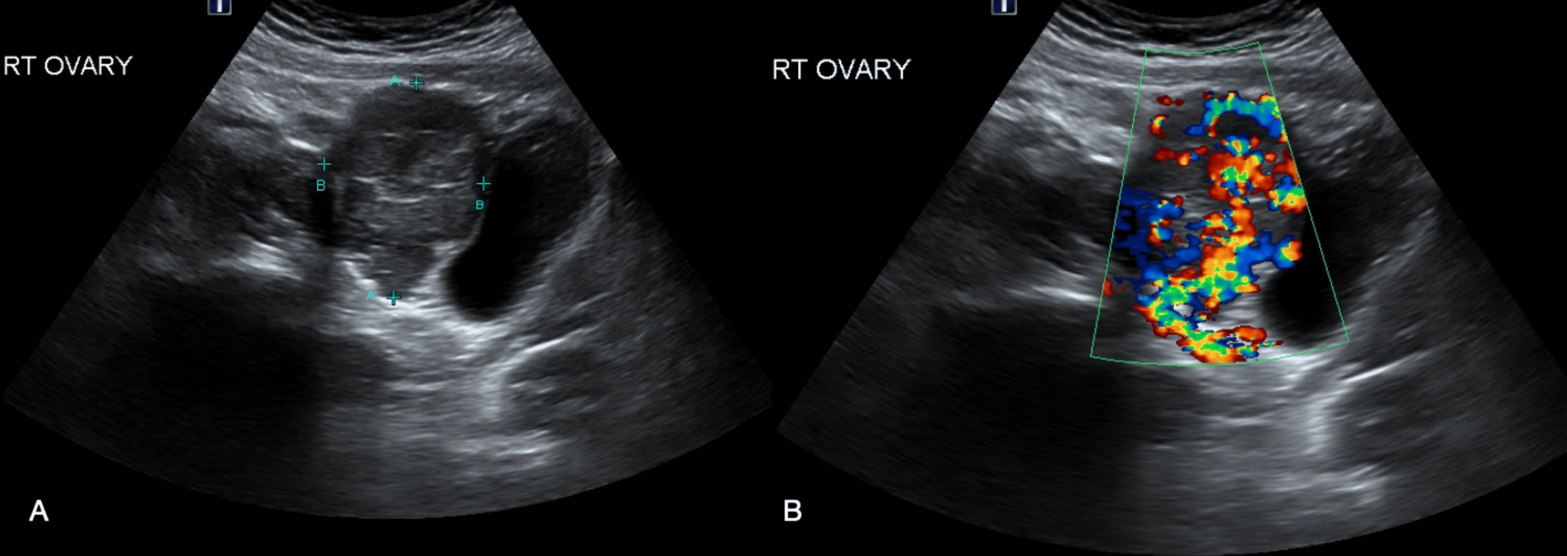
Ultrasound (pelvis) shows (A) well-defined, solid hypoechoic mass in the right adnexa and (B) hypervascularity on color doppler imaging.

We present a case of a 16-year-old female patient who was admitted for hypertensive urgency and worsening left-sided chest pain, recurrent headaches, and 10 lbs of unintentional weight loss within one month. The patient had poorly controlled hypertension despite being on lisinopril and metoprolol. Laboratory tests to rule out secondary causes of hypertension, including thyroid function tests, serum renin, and aldosterone, yielded no abnormalities. However, plasma metanephrines were elevated at 4463.4 pmol/L (normal range <510 pmol/L), indicating an underlying catecholamine-secreting tumor. Abdominal ultrasound (Figure 1) revealed a normal sonographic appearance of the kidneys but identified a hypervascular mass in the right adnexa.

**Figure 2:**
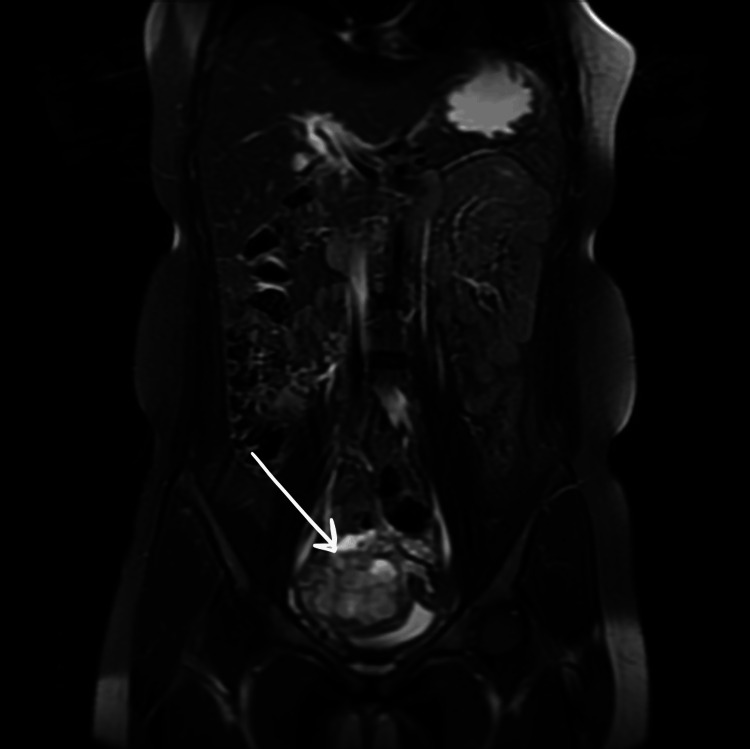
Coronal MRI (abdomen and pelvis) T2 fat-saturated sequence shows hyperintense lobulated soft tissue mass in the right hemipelvis arising from the urinary bladder.

MRI of the abdomen and pelvis (Figure 2) showed an enhanced T2 hyperintense lobulated soft tissue mass in the right hemipelvis with diffusion restriction, which appeared to be arising from the urinary bladder. 

**Figure 3:**
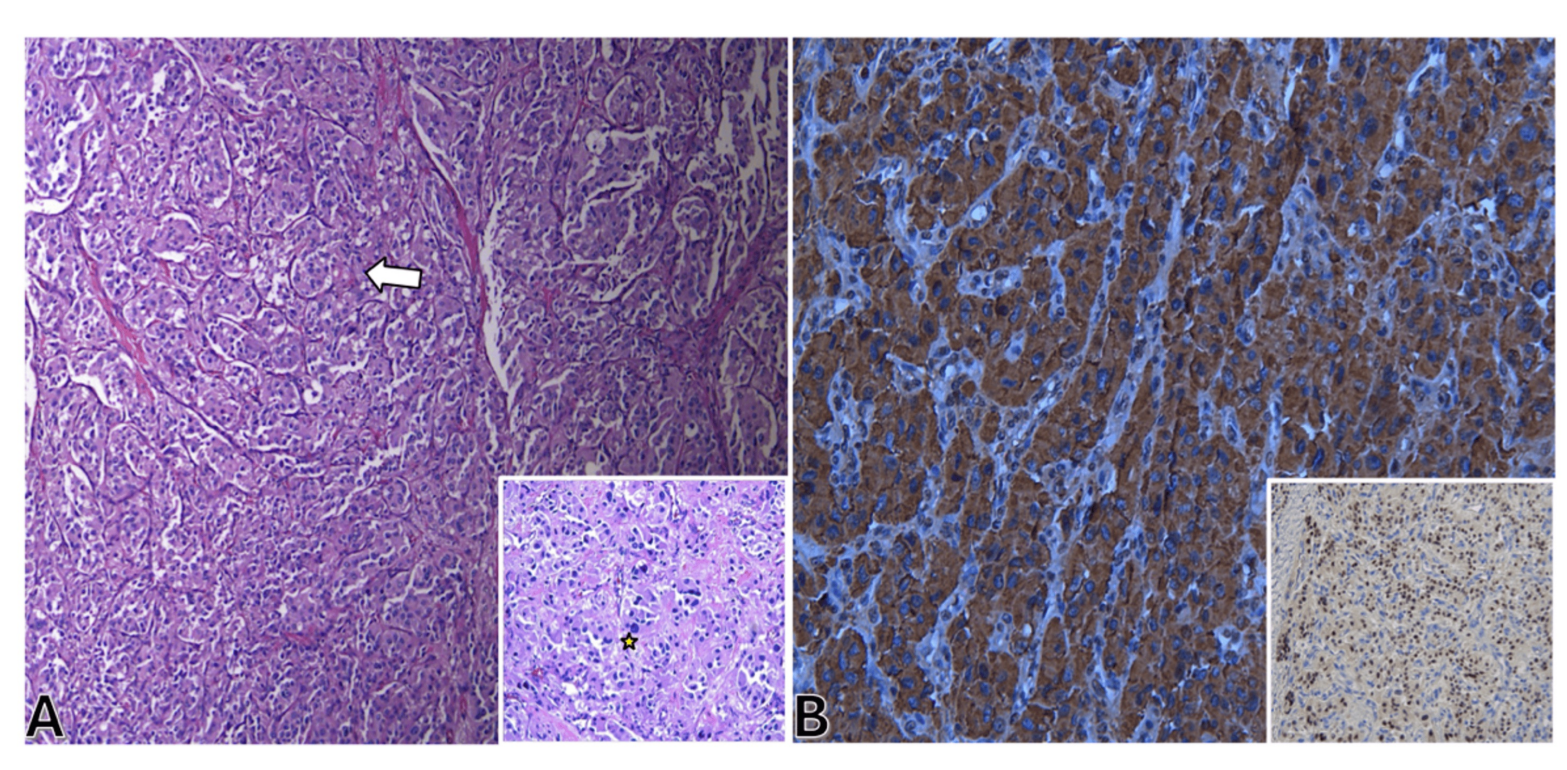
Microscopic images of the bladder specimen. (A) H&E stain of bladder pheochromocytoma (x40). The zellballen (nested) pattern of chromaffin cells present (arrow) is pathognomonic for pheochromocytoma. Inset: H&E stain shows mild, patchy hyperchromatic, pleomorphic chromaffin cells (star) (x100). (B) Diffuse immunoreactivity for chromogranin A (neuroendocrine cell marker) in the neoplastic cells supports the diagnosis (x100). Inset: Nuclear immunoreactivity for GATA3 (epithelial neoplasm marker) is seen in 95% of the nuclei, ruling out adrenal cortical neoplasm (x100). H&E, hematoxylin and eosin

Based on the imaging findings, extra-adrenal pheochromocytoma/paraganglioma was the leading consideration. The patient was started on phenoxybenzamine along with metoprolol, and lisinopril was discontinued. During surgery, the tumor was discovered to involve the right posterolateral bladder wall and was resected with a partial cystectomy. Histopathology with H&E stain (Figure 3) showed hyperchromatic, pleomorphic chromaffin cells and diffuse immunoreactivity for chromogranin A, consistent with pheochromocytoma/paraganglioma.

## Discussion

Pheochromocytoma is seen in less than 0.2% of hypertensive patients, with urinary bladder pheochromocytoma accounting for less than 1% of all cases [5]. Pheochromocytoma usually presents with the triad of sustained hypertension, headache, and palpitations/chest pain. In addition, patients with urinary bladder pheochromocytoma present with hematuria or symptomatic blood pressure fluctuations during micturition, bladder distension, and bladder procedures such as cystoscopic biopsy [5]. These lesions are commonly associated with familial syndromes including neurofibromatosis, von Hippel-Lindau, Sturge-Weber, tuberous sclerosis, and multiple endocrine neoplasia type II [6].

Patients with pheochromocytoma have significantly elevated plasma and urinary metanephrines, indicating an underlying catecholamine-secreting tumor. If suspected clinically and based on laboratory values, imaging should be the next step in management to localize the tumor and any possible sites of metastasis. Currently, CT is the most commonly used initial imaging investigation to evaluate patients with suspected bladder tumors; however, few studies have shown the importance of MRI in such cases [4]. MRI is more sensitive and provides improved soft tissue contrast, leading to more precise staging [7], and is preferred over CT in the pediatric population due to the obvious benefit of avoiding radiation exposure. Pheochromocytoma demonstrates an intermediate signal on T1 and T2 hyperintensity due to high water content and rich vascularity [6]. 

The histological results show sustentacular cells of pheochromocytomas exhibiting immunoreactivity to the S-100 protein, which closely resembles the chief cells of typical adrenal medulla, displaying an immune response to CD56, chromogranin A, and synaptophysin [8].

Management of bladder pheochromocytoma involves both surgical and medical methods. The most frequent surgical procedure is partial cystectomy, while radical cystectomy with pelvic lymph node dissection is done for malignant diseases. Preoperative administration of α and β receptor blocking agents is essential to prevent the occurrence of hypertensive crisis during surgery [7]. 

For individuals with malignant pheochromocytoma, it is advised to monitor blood pressure and have yearly postoperative surveillance. Metaiodobenzylguanidine imaging (MIBG) can also be utilized in cases with high suspicion of recurrence or incomplete resection since it is more specific, particularly in identifying adrenergic tissue or metastatic disease [6].

## Conclusions

A combination of specific symptoms, laboratory tests, histology from tissue biopsy, and image investigation is critical to the diagnosis of urinary bladder pheochromocytoma. Imaging studies can help localize the tumor. Surgical resection is the treatment of choice, and careful monitoring and control of blood pressure with medical management are critical to avoid a hypertensive crisis during surgery. Long-term follow-up is recommended in cases of malignant tumors to monitor for recurrence.
